# Duodenal tuberculosis with probable biliary involvement in primary Sjögren’s syndrome mimicking pancreatobiliary malignancy: a case report

**DOI:** 10.3389/fimmu.2026.1874119

**Published:** 2026-07-08

**Authors:** Jing Zhang, Xucheng Zhou, Sheng-Guang Li, Lina Zhang, Wenzai Shi

**Affiliations:** 1 Department of Rheumatology and Immunology, Peking University International Hospital, Beijing, China; 2 Department of Geriatrics, Guilin People’s Hospital, Guilin, China; 3 Department of General Surgery, Peking University International Hospital, Beijing, China

**Keywords:** duodenal tuberculosis, extrapulmonary tuberculosis, fistula, lymphoma, malignancy mimic, primary sjögren’s syndrome, probable biliary involvement

## Abstract

**Background:**

Extrapulmonary tuberculosis can imitate malignancy and systemic autoimmune disease. Concurrent duodenal tuberculosis with probable biliary involvement is exceptionally uncommon and may create a high-stakes pancreatobiliary cancer mimic.

**Case presentation:**

A 71-year-old Han Chinese woman with primary Sjögren’s syndrome and type 2 diabetes, and no recent immunosuppressive therapy, presented with polyarthralgia, intermittent fever, abdominal discomfort, anorexia, constipation, and weight loss. Laboratory testing showed systemic inflammation and a cholestatic enzyme pattern, whereas bilirubin and tumor markers remained normal. Magnetic resonance cholangiopancreatography revealed diffuse bile-duct wall thickening with severe distal common bile duct stenosis, and positron emission tomography-computed tomography showed hypermetabolic duodenal thickening with multiple fluorodeoxyglucose-avid lymph nodes. The tuberculin skin test measured 15 x 12 mm at 48 and 72 hours, and interferon-gamma release assay was positive (T-N >10.00 IU/mL). Upper endoscopy demonstrated a duodenal ulcer with fistula formation, and biopsy showed ulceration, granulomatous inflammation, and one acid-fast bacillus on staining. Supraclavicular lymph-node aspiration revealed lymphocytes and multinucleated giant cells without malignant cells. Duodenal tuberculosis with nodal disease and probable, non-biopsy-proven biliary involvement was diagnosed. After discharge, she received specialist-supervised antituberculosis therapy including isoniazid 0.3 g daily, rifampicin 0.45 g daily, and levofloxacin 0.5 g daily; by January 2022, treatment had continued for more than 3 months. The initial phase structure and total treatment duration could not be reliably ascertained. Symptoms, inflammatory and cholestatic markers, endoscopic ulceration, fistula, lymph-node size, and common bile duct stenosis improved at reassessment in January 2022.

**Conclusion:**

Tuberculosis should remain in the differential diagnosis of fluorodeoxyglucose-avid duodenal and biliary lesions in patients with autoimmune disease, particularly when biochemical obstruction, tumor markers, and tissue pathology do not fit malignancy. Early endoscopic biopsy and accessible nodal sampling can prevent unnecessary surgery and unsafe escalation of immunosuppression.

## Introduction

Tuberculosis remains a major infectious cause of morbidity worldwide, and extrapulmonary disease continues to create diagnostic difficulty because it may involve almost any organ system (1, 2). Abdominal tuberculosis is particularly deceptive: constitutional symptoms, abdominal pain, weight loss, lymphadenopathy, and fluorodeoxyglucose (FDG) uptake can overlap with malignancy, inflammatory bowel disease, and autoimmune hepatobiliary disease (3–5). Because gastrointestinal tuberculosis frequently presents with nonspecific constitutional and abdominal symptoms and lacks a single definitive diagnostic test, diagnosis often depends on integrating clinical suspicion, imaging, endoscopy, microbiological testing, histopathology, multidisciplinary assessment, and therapeutic response (3–5).

Foregut tuberculosis is far less common than ileocecal or peritoneal disease, and duodenal involvement may present with ulceration, obstruction, bleeding, perforation, or fistula formation (6–9). Biliary tuberculosis is rarer still, but reported presentations include bile-duct stricture, obstructive jaundice, and cholangiocarcinoma-like imaging (10–13).

Primary Sjögren’s syndrome (pSS) adds a further layer of ambiguity. Systemic symptoms, arthralgia, abnormal liver enzymes, and hypergammaglobulinemia may direct clinicians toward autoimmune activity or overlap disease, whereas older age, diabetes, immune dysregulation, and immunomodulatory therapy when present require vigilance for infection. A nationwide cohort study found a higher risk of tuberculosis in patients with pSS than in matched controls (14). We describe a patient with pSS in whom duodenal tuberculosis with nodal disease and probable biliary involvement mimicked pancreatobiliary malignancy. The case highlights diagnostic lessons relevant to rheumatologists, gastroenterologists, surgeons, radiologists, infectious disease clinicians, and hematologists.

## Case description

A 71-year-old Han Chinese woman from Beijing with a farming occupation was admitted to the rheumatology department in August 2021 with 20 days of worsening fatigue, anorexia, diffuse musculoskeletal pain, abdominal discomfort, constipation, intermittent fever, and an approximately 3.5-kg weight loss. She had long-standing intermittent oral and ocular dryness and progressive dental loss. Decades earlier, she had experienced recurrent generalized pain and had received oral prednisone for a presumed rheumatic disorder; glucocorticoids were stopped after approximately 1 year because of adverse effects. Her medical history included type 2 diabetes, osteoporosis, and lumbar disc disease. She had no recent immunosuppressive therapy before admission, no tuberculosis contact history, no prior pulmonary tuberculosis, and no residence in a known infectious or endemic disease area. BCG vaccination status, HIV status, and formal immunodeficiency evaluation were unavailable.

At admission, temperature was 37.1 °C, heart rate 71 beats/min, respiratory rate 19 breaths/min, and blood pressure 113/53 mmHg; oxygen saturation was unavailable. Physical examination showed no jaundice, palpable superficial lymphadenopathy, hepatosplenomegaly, abdominal mass, peritoneal signs, or mucocutaneous lesions. The abdomen was soft without tenderness or rebound. The shoulders, elbows, hips, and knees were tender without obvious swelling, and 12 fibromyalgia tender points were positive. Mild epigastric or right-upper-quadrant tenderness appeared intermittently during follow-up.

Initial laboratory tests showed hemoglobin 117 g/L, erythrocyte sedimentation rate (ESR) 58 mm/h, C-reactive protein (CRP) 11.61 mg/L, albumin 30.9 g/L, alanine aminotransferase 55 U/L, aspartate aminotransferase 44 U/L, alkaline phosphatase (ALP) 514 U/L, and gamma-glutamyl transferase (GGT) 354 U/L. Total and direct bilirubin were normal. Renal function, coagulation studies, urinalysis, and stool testing did not explain the presentation. Tumor markers, including carbohydrate antigen 19-9, carcinoembryonic antigen, and alpha-fetoprotein, were within the reference range.

**Figure 1:**
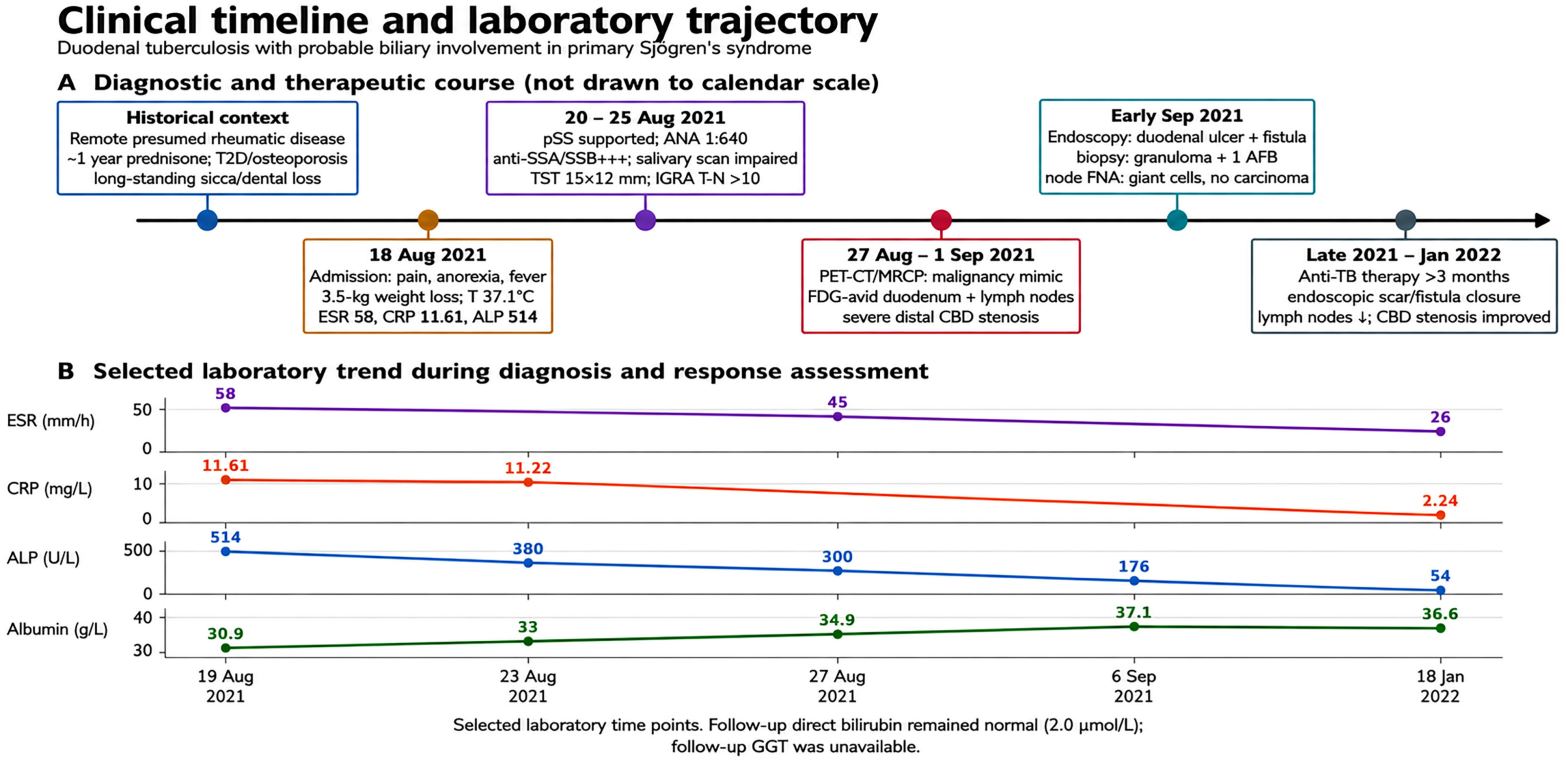
Clinical timeline and selected laboratory trajectory of the diagnostic episode. **(A)** shows the transition from long-standing sicca and autoimmune context to malignancy-like pancreatobiliary imaging, tissue-supported duodenal tuberculosis diagnosis, and objective response after antituberculosis therapy. **(B)** shows selected inflammatory, cholestatic, and nutritional laboratory values over time. Direct bilirubin remained normal at baseline and follow-up; follow-up GGT was unavailable. AFB, acid-fast bacillus; ALP, alkaline phosphatase; CBD, common bile duct; CRP, C-reactive protein; ESR, erythrocyte sedimentation rate; FDG, fluorodeoxyglucose; GGT, gamma-glutamyl transferase; IGRA, interferon-gamma release assay; MRCP, magnetic resonance cholangiopancreatography; PET-CT, positron emission tomography-computed tomography; pSS, primary Sjögren’s syndrome; TST, tuberculin skin test.

Immunological testing showed antinuclear antibody 1:640, strongly positive anti-SSA/Ro60, anti-SSA/Ro52, and anti-SSB/La antibodies, elevated IgG, and negative rheumatoid factor, anti-cyclic citrullinated peptide antibody, antimitochondrial antibody, AMA-M2, and antineutrophil cytoplasmic antibodies. The combination of long-standing sicca symptoms, positive Schirmer testing, impaired uptake and excretory function on salivary gland scintigraphy, and anti-SSA positivity supported pSS according to the 2016 ACR/EULAR classification criteria (15). The episode of care, including selected laboratory trajectories, is summarized in **Figures 1A, B**.

## Diagnostic assessment

**Figure 2:**
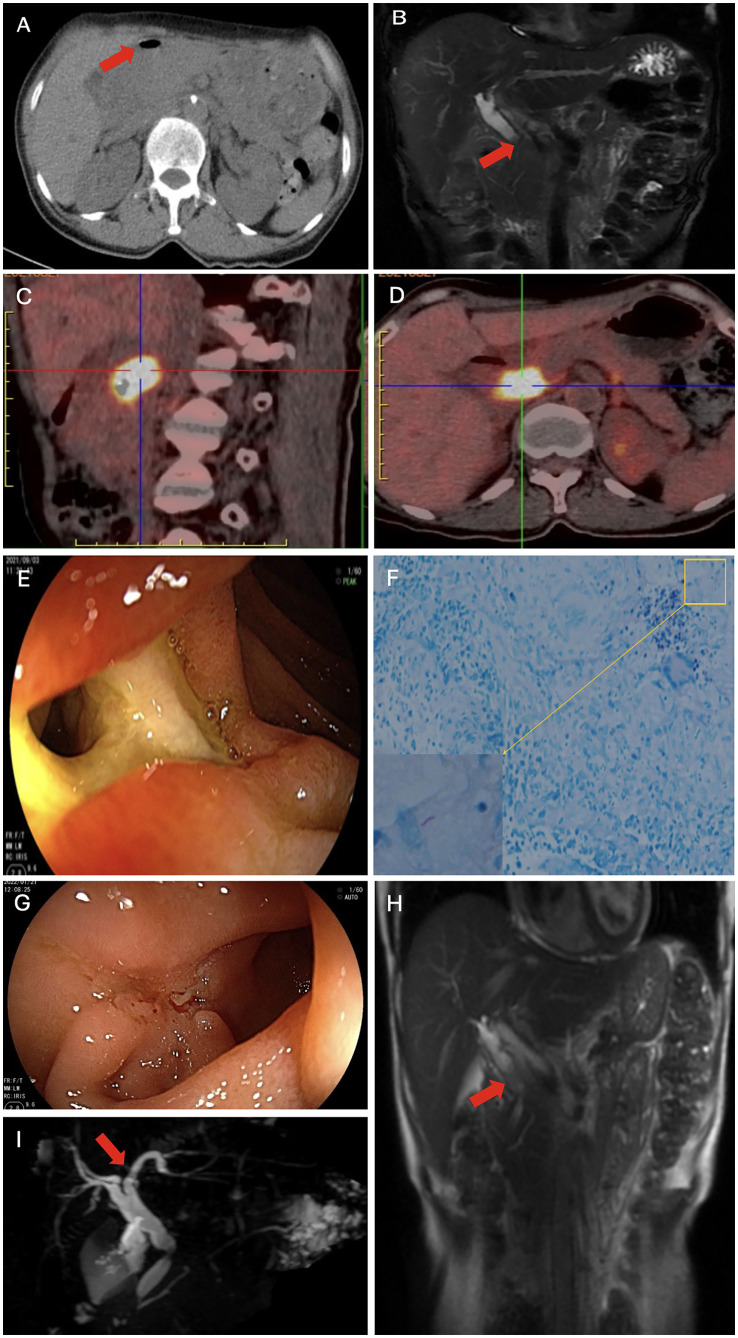
Multimodal findings of duodenal tuberculosis with probable biliary involvement and response to therapy. Red arrows mark the relevant abnormality in each panel; PET-CT crosshairs identify FDG-avid lesions. **(A)** Baseline abdominal CT showing a small oval gas density near the left intrahepatic bile duct/duodenal region, suggesting a tiny, contained perforation or fistulous process. **(B)** Baseline MRI/MRCP demonstrating distal common bile duct stenosis. **(C, D)** PET-CT showing hypermetabolic duodenal and peripancreatic/nodal lesions, initially concerning for malignancy. **(E)** Upper endoscopy showing duodenal ulceration with fistula formation. **(F)** Acid-fast staining of duodenal biopsy showing one acid-fast bacillus in the lesion. Magnification was unavailable. **(G)** Follow-up endoscopy after antituberculosis therapy showing healing and fistula closure. **(H, I)** Follow-up MRCP/MRI showing improved common bile duct stenosis and periampullary inflammatory changes.

Abdominal computed tomography (CT) showed small intrahepatic bile-duct gas and a full pancreatic contour (**Figure 2A**). Contrast-enhanced magnetic resonance imaging (MRI) and magnetic resonance cholangiopancreatography (MRCP) demonstrated mild intrahepatic and common bile duct dilatation, diffuse bile-duct wall thickening, severe distal common bile duct stenosis, and enlarged hepatoduodenal and peripancreatic lymph nodes (**Figure 2B**). Positron emission tomography-computed tomography (PET-CT) showed hypermetabolic thickening of the duodenal wall and FDG-avid lymph nodes adjacent to the pancreatic head, behind the pancreas, along the abdominal aorta, beside the iliac vessels, and in the left supraclavicular region (**Figures 2C, D**). This pattern initially suggested duodenal, pancreatobiliary, or lymphoproliferative malignancy with nodal involvement.

The mismatch between severe-appearing biliary narrowing and normal bilirubin, normal tumor markers, and constitutional inflammatory symptoms prompted parallel evaluation for infection. The tuberculin skin test measured 15 x 12 mm at 48 hours and remained 15 x 12 mm at 72 hours. Interferon-gamma release assay was positive (T-N >10.00 IU/mL). Pulmonary tuberculosis was assessed clinically and radiographically: the patient had no cough or sputum production, a chest radiograph before admission showed no active pulmonary disease, and chest CT/PET-CT during the diagnostic admission showed scattered micronodules or mild inflammatory/old changes rather than a dominant active pulmonary focus. Sputum mycobacteriology was not obtained. Upper endoscopy showed a duodenal ulcer with fistula formation at the junction of the duodenal bulb and descending duodenum (**Figure 2E**). Duodenal biopsy demonstrated ulceration, granulation tissue, granulomatous inflammation, and one acid-fast bacillus on staining (**Figure 2F**). Fine-needle aspiration of a supraclavicular lymph node showed abundant lymphocytes and multinucleated giant cells without malignant cells.

A lesion-directed acid-fast stain strongly supported mycobacterial infection, but mycobacterial culture, nucleic acid amplification testing, Xpert MTB/RIF, and drug-susceptibility testing were not obtained from duodenal tissue, lymph node material, sputum, or another specimen. This limited species-level confirmation and drug-susceptibility assessment. Given pSS, weight loss, constitutional symptoms, and FDG-avid lymphadenopathy, lymphoma was also considered. The supraclavicular-node aspirate was reassuring for the absence of overt malignant cells, and subsequent nodal regression after antituberculosis therapy made lymphoma less likely for this episode; however, flow cytometry, core biopsy, and excisional biopsy were not obtained, and fine-needle aspiration alone is not equivalent to definitive lymphoma exclusion in all clinical contexts.

Taken together, the findings supported duodenal tuberculosis with nodal involvement and probable biliary involvement. The biliary component was considered probable rather than biopsy-proven because bile-duct tissue was not sampled; it was supported by the imaging pattern, adjacent duodenal and nodal tuberculosis, absence of malignant cells in the sampled node, and subsequent radiologic and biochemical response to antituberculosis therapy. Key features supporting this diagnostic interpretation are summarized in **Table 1**.

**Table 1:** Key disease features supporting the final diagnostic interpretation.

Feature domain	Main findings in this case	Diagnostic implication
Host context	pSS, older age, type 2 diabetes, Han Chinese ethnicity, Beijing farming occupation, remote historical prednisone exposure, and no recent immunosuppression.	Creates autoimmune-infectious-malignant diagnostic ambiguity while distinguishing remote steroid exposure from proximate immunosuppressive risk.
Clinical syndrome	Fatigue, anorexia, intermittent fever, diffuse pain, abdominal discomfort, constipation, and 3.5-kg weight loss; initial examination lacked jaundice, peritoneal signs, organomegaly, or palpable lymphadenopathy.	Constitutional and abdominal symptoms overlapped with tuberculosis, malignancy, lymphoma, and autoimmune flare.
Biochemical pattern	ESR/CRP elevated; ALP and GGT markedly elevated; bilirubin and tumor markers normal.	Cholestatic injury was present, but normal bilirubin and tumor markers were discordant with advanced malignant obstruction; they reduced but did not exclude malignancy.
Autoimmune work-up	ANA 1:640, anti-SSA/Ro and anti-SSB/La positive; Schirmer test and salivary scintigraphy supportive; AMA/AMA-M2 and ANCA negative.	Supported pSS while weakening the case for primary biliary cholangitis or ANCA-associated hepatobiliary disease.
Imaging	MRCP showed distal common bile duct stenosis; PET-CT showed FDG-avid duodenal thickening and multiple lymph nodes.	A strong malignancy and lymphoma mimic that required tissue confirmation rather than imaging-based surgery.
Tuberculosis tests	TST 15 x 12 mm at 48 and 72 hours; IGRA positive with T-N >10.00 IU/mL.	Supported tuberculosis exposure/infection but could not alone prove active disease.
Tissue evidence	Duodenal ulcer with fistula; biopsy showed granulomatous inflammation and one acid-fast bacillus; supraclavicular node FNA showed giant cells without malignant cells.	Established lesion-level mycobacterial disease and argued against metastatic carcinoma in the sampled node, while not fully excluding lymphoma by FNA alone.
Treatment response	After antituberculosis therapy, symptoms improved, ESR/CRP/ALP normalized or markedly decreased, albumin increased, fistula closed, lymph nodes regressed, and common bile duct stenosis improved.	Therapeutic response supported the integrated diagnosis, including probable biliary involvement.

This table summarizes the convergent clinical, biochemical, autoimmune, imaging, microbiological, pathological, and therapeutic-response evidence supporting duodenal tuberculosis with nodal disease and probable biliary involvement, while highlighting competing diagnostic considerations including malignancy, lymphoma, and autoimmune hepatobiliary disease. AFB, acid-fast bacillus; ALP, alkaline phosphatase; AMA, antimitochondrial antibody; AMA-M2, antimitochondrial antibody M2 subtype; ANA, antinuclear antibody; ANCA, antineutrophil cytoplasmic antibody; CRP, C-reactive protein; ESR, erythrocyte sedimentation rate; FDG, fluorodeoxyglucose; FNA, fine-needle aspiration; GGT, gamma-glutamyl transferase; IGRA, interferon-gamma release assay; MRCP, magnetic resonance cholangiopancreatography; PET-CT, positron emission tomography-computed tomography; pSS, primary Sjögren’s syndrome; TST, tuberculin skin test.

## Therapeutic intervention

After multidisciplinary discussion, surgery for presumed malignancy was deferred and the patient was referred for specialist tuberculosis management. Specialist-guided multidrug antituberculosis therapy was initiated after discharge. By January 2022, she had received more than 3 months of isoniazid 0.3 g daily, rifampicin 0.45 g daily, and levofloxacin 0.5 g daily, with continued monitoring of symptoms and liver biochemistry. The exact treatment start date, complete initial regimen, intensive- and continuation-phase structure, total planned duration, and total completed duration were unavailable. Pyrazinamide and ethambutol could not be confirmed as part of the regimen; no rationale for their omission was inferred.

Treatment adherence and tolerability were monitored through specialist follow-up, interval clinical reassessment, medication reconciliation, and serial liver biochemistry. No treatment-limiting adverse drug reaction emerged during early follow-up; liver enzymes had normalized by January 2022. Nutritional support and avoidance of unnecessary immunosuppression were emphasized while active infection was being treated. Hydroxychloroquine 0.2 g twice daily was introduced later for persistent Sjögren-related arthralgia only after clinical response to antituberculosis therapy had become evident. The pharmacologic and monitoring information is summarized in **Table 2**.

**Table 2:** Medication and monitoring information.

Medication	Dose	Timing/role	Monitoring notes
Isoniazid	0.3 g orally once daily	Specialist-guided antituberculosis therapy; more than 3 months had been completed by January 2022	Phase structure and total duration were unavailable; liver function and symptoms were monitored.
Rifampicin	0.45 g orally once daily	Specialist-guided antituberculosis therapy; more than 3 months had been completed by January 2022	Phase structure and total duration were unavailable; liver function and symptoms were monitored.
Levofloxacin	0.5 g orally once daily	Specialist-guided antituberculosis therapy; more than 3 months had been completed by January 2022	Used under specialist tuberculosis management; the indication for a fluoroquinolone-containing regimen was unclear.
Pyrazinamide/ethambutol	Use not confirmed	Dose, phase, and duration unavailable	No clinical rationale for use or omission was inferred. Treatment decisions were made by a specialist tuberculosis service.
Hydroxychloroquine	0.2 g orally twice daily	Introduced later for persistent Sjögren-related arthralgia after objective response to antituberculosis therapy	Introduced at January 2022 discharge after infection control was prioritized.
Cefdinir	0.1 g orally three times daily	Short course during January 2022 follow-up for suspected bacterial pneumonia	Not part of tuberculosis treatment; included for medication transparency.

This table lists the antituberculosis and adjunctive medications documented during follow-up, including available doses, timing or clinical role, and monitoring considerations. The table also clarifies unavailable regimen details, including the unconfirmed use of pyrazinamide and ethambutol.

## Follow-up and outcomes

At reassessment in January 2022, after more than 3 months of antituberculosis therapy, abdominal discomfort and systemic symptoms had improved. Laboratory indices on 18 January showed parallel improvement: ESR decreased from 58 to 26 mm/h, CRP from 11.61 to 2.24 mg/L, and ALP from 514 to 54 U/L, while albumin increased from 30.9 to 36.6 g/L (**Figure 1B**). Direct bilirubin remained normal at 2.0 µmol/L; GGT was not reassessed.

At the same reassessment, lymph-node ultrasonography showed marked reduction of the supraclavicular node. Endoscopy demonstrated healing of the duodenal lesion with scar formation and no persistent fistula (**Figure 2G**), and MRI/MRCP showed marked improvement of the distal common bile duct stenosis and peripancreatic lymphadenopathy compared with the September 2021 evaluation (**Figures 2H, I**). These objective responses strengthened the integrated diagnosis and avoided pancreatobiliary surgery. No relapse, recurrent biliary stenosis, recurrent fistula, or treatment-related complication emerged during the available early follow-up period, although longer-term follow-up was unavailable.

## Discussion

This case combines three uncommon and easily misread elements: foregut tuberculosis, probable biliary involvement, and pSS. The initial presentation strongly resembled malignancy. PET-CT demonstrated FDG-avid duodenal thickening and widespread lymphadenopathy, while MRCP showed severe distal bile duct stenosis. Normal bilirubin and tumor markers made advanced obstructive pancreatobiliary malignancy less typical but did not exclude cancer. The decisive diagnostic step was tissue acquisition from the duodenal ulcer and an accessible lymph node, followed by objective response assessment.

Duodenal tuberculosis is rare because gastric acidity, rapid transit, and relatively sparse lymphoid tissue in the upper gastrointestinal tract are thought to reduce mycobacterial implantation (3, 6–8). Recent reports continue to describe gastroduodenal and pyloroduodenal tuberculosis as a mimic of gastric outlet obstruction or malignancy, often requiring endoscopic biopsy, endoscopic ultrasound-guided sampling, or surgical pathology for diagnosis (6–8). Fistula formation is especially unusual; a prior report described duodenal tuberculosis with a choledochoduodenal fistula (9). Our patient had a duodenal fistula without acute peritonitis, suggesting a chronic contained process.

The biliary lesion in this case should be described cautiously. Hepatobiliary and biliary tuberculosis may cause bile-duct strictures and cholangiocarcinoma-like imaging (10–13), but direct bile-duct biopsy was not performed in this patient. The designation of probable biliary involvement is therefore based on convergent evidence: severe distal common bile duct stenosis, adjacent duodenal and nodal tuberculosis, absence of malignant cells in the sampled node, and marked radiologic and biochemical improvement after antituberculosis therapy. This terminology preserves the clinical novelty of the case while avoiding overinterpretation of an unbiopsied biliary lesion.

The differential diagnosis was broader than carcinoma. Duodenal Crohn’s disease, gastrointestinal histoplasmosis, duodenal gastrointestinal stromal tumor, lymphoma, IgG4-related disease, primary biliary cholangitis, and other inflammatory or malignant pancreatobiliary disorders can produce overlapping symptoms, ulceration, wall thickening, or obstructive imaging patterns (16–19). In this patient, AMA/AMA-M2 and ANCA were negative, IgG4-related disease was not supported by the clinical, serological, and imaging profile, and normal bilirubin was atypical for advanced obstructive malignancy. However, pSS is associated with an increased risk of non-Hodgkin lymphoma (19). Therefore, FDG-avid lymphadenopathy and weight loss in pSS should not be attributed to infection or autoimmune activity without tissue evaluation. The lymph-node aspirate and subsequent regression were reassuring in this episode, but the absence of flow cytometry, core biopsy, or excisional biopsy limits the certainty with which lymphoma can be excluded.

The pSS context matters for clinical decision-making. pSS may distract attention toward autoimmune hepatobiliary overlap when cholestatic enzymes are present. Conversely, population-based data suggest that patients with pSS have an increased risk of tuberculosis (14). Mechanisms may include immune dysregulation, older age, comorbid diabetes, and immunomodulatory therapy when present. In this case, glucocorticoid exposure occurred decades earlier and was treated as historical autoimmune context rather than a proximate driver of reactivation. Systemic immunomodulatory escalation was deferred while infection and malignancy were evaluated and treated. Persistent fever, weight loss, FDG-avid nodes, and atypical gastrointestinal lesions should prompt tissue-based infection and malignancy work-up before escalation of immunosuppression.

Interferon-gamma release assays and tuberculin skin tests are supportive but cannot distinguish latent infection from active disease (20). The diagnostic weight in this case came from lesion-directed biopsy showing acid-fast bacilli, compatible histopathology, nodal cytology, and objective therapeutic response. Long-term experience with abdominal tuberculosis similarly emphasizes that diagnosis often requires combining clinical suspicion, imaging, endoscopy, pathology, microbiology, and response to treatment (21). WHO guidance on drug-susceptible tuberculosis underscores the need for regimen selection and duration under appropriate tuberculosis expertise (22). The focused literature context is summarized in **Table 3**.

**Table 3:** Focused literature context for this case.

Literature source	Relevant pattern	Relevance to this case
Gastrointestinal and foregut tuberculosis (3–8, 21)	Nonspecific constitutional and abdominal symptoms; limited value of any single diagnostic test; foregut disease may mimic obstruction or malignancy.	Supports an integrated diagnosis based on imaging, endoscopy, pathology, microbiology when available, and response to treatment.
Duodenal fistula and biliary adjacency (9)	Duodenal tuberculosis with choledochoduodenal fistula has been reported.	Provides a close historical analogue to this patient’s duodenal fistula and biliary-adjacent disease.
Hepatobiliary/biliary tuberculosis (10–13)	Hepatobiliary tuberculosis and common bile duct strictures can mimic cholangiocarcinoma or pancreatobiliary cancer.	Frames the distal common bile duct stenosis as a recognized but rare tuberculosis-related pattern.
Duodenal Crohn’s disease, histoplasmosis, and duodenal GIST (16–18)	Non-tuberculous duodenal inflammatory, infectious, and neoplastic diseases may mimic ulcerating or mass-like duodenal disease.	Broadens the differential diagnosis beyond carcinoma and supports tissue acquisition.
pSS, tuberculosis, and lymphoma (14, 19)	pSS has been associated with increased tuberculosis risk and non-Hodgkin lymphoma risk.	Justifies vigilance for both infection and lymphoma before immunosuppression.
Tuberculosis testing and treatment guidance (20, 22)	TST/IGRA support infection/exposure but cannot by themselves prove active disease; treatment should be individualized under tuberculosis expertise.	Explains the diagnostic role of immunologic tests and the need for specialist-guided therapy and monitoring.

This table places the case in the context of focused literature on gastrointestinal and foregut tuberculosis, duodenal fistula, hepatobiliary or biliary tuberculosis, non-tuberculous duodenal mimics, pSS-associated tuberculosis and lymphoma risk, and tuberculosis testing and treatment guidance. It supports the integrated diagnostic interpretation and the cautious use of “probable” for biliary involvement. GIST, gastrointestinal stromal tumor; IGRA, interferon-gamma release assay; pSS, primary Sjögren’s syndrome; TST, tuberculin skin test.

This report has limitations. BCG vaccination status, HIV status, formal immunodeficiency evaluation, exact Schirmer values, quantitative salivary scintigraphy metrics, sputum mycobacteriology, mycobacterial culture, nucleic acid confirmation, Xpert MTB/RIF, drug-susceptibility results, complete initial treatment regimen, treatment phase structure, total treatment duration, follow-up GGT values, and longer-term follow-up were unavailable. The biliary lesion was not biopsied. Fine-needle aspiration of one lymph node was reassuring but is less definitive than core or excisional biopsy with flow cytometry for excluding lymphoma in some contexts.

In conclusion, duodenal tuberculosis with probable biliary involvement can masquerade as pancreatobiliary malignancy in pSS. In patients with autoimmune disease, constitutional symptoms, FDG-avid lymphadenopathy, and discordant cholestatic findings, clinicians should obtain endoscopic and nodal tissue early and avoid anchoring on malignancy or autoimmune flare alone.

## Data Availability

The de-identified clinical data supporting this case report are included within the article. Additional de-identified information can be made available by the corresponding author on reasonable request, subject to institutional and patient-consent requirements.
